# Metabolic Regulation of Influenza Vaccine Responses in Racially Diverse Hispanics

**DOI:** 10.3390/vaccines13090938

**Published:** 2025-09-02

**Authors:** Daniela Frasca, Maria Romero, Suresh Pallikkuth

**Affiliations:** 1 Department of Microbiology and Immunology, University of Miami Miller School of Medicine, Miami, FL 33136, USA; mromero5@med.miami.edu; 2 Sylvester Comprehensive Cancer Center, University of Miami Miller School of Medicine, Miami, FL 33136, USA

**Keywords:** health disparities, humoral immunity, influenza vaccine

## Abstract

Background: Racial and ethnic differences in vaccine responses, particularly within Hispanic populations, remain underexplored. Disparities in immune function may be influenced by metabolic and inflammatory mechanisms. Methods: The current study investigated humoral immune responses to influenza vaccination in a diverse cohort of Hispanic individuals from South Florida, encompassing both White and Black Hispanics. Antibody responses were assessed post-vaccination, and B cell phenotypes were analyzed to evaluate inflammatory and metabolic characteristics. In vitro experiments were conducted to determine whether blocking metabolic pathways could alter the inflammatory phenotype of B cells. Data were analyzed using an unpaired Student’s *t*-test (two-tailed), and correlation analysis was conducted with Pearson correlation. Results: Our findings indicated that Black Hispanic individuals exhibited significantly reduced antibody responses compared to White Hispanics (*p* < 0.01) following influenza vaccination. This diminished humoral response correlated with inversely with serum LDH (r = −0.58; *p* = 0.0005) and other intrinsic inflammatory phenotypes in blood-derived B cells and was supported by changes in metabolic activity. In vitro blockade of metabolic pathways effectively reduced the inflammatory phenotype of B cells from Black Hispanic individuals, suggesting a mechanistic link between metabolic dysfunction and impaired vaccine-induced immunity. Conclusion: This study is the first to reveal racial disparities in influenza vaccine responses within a Hispanic population, highlighting reduced antibody production in Black Hispanics. These findings suggest that metabolically driven B cell inflammation may play a critical role and point to potential therapeutic strategies to address disparities in vaccine-induced immunity.

## 1. Introduction

Immunity and its impact on racial and ethnic health disparities is a complex and multifactorial issue, influenced by various factors that affect immune cell function and health outcomes among diverse groups [1]. Equitable immunization strategies not only help to prevent vaccine-preventable diseases but also promote overall health by encouraging people, including marginalized groups, to engage with healthcare, thereby addressing other health inequalities [2]. Racial and ethnic minorities frequently often experience chronic stress stemming from systemic racism, discrimination, and socioeconomic challenges, which can dysregulate immune function. This immune dysregulation may lead to increased inflammation and a reduced ability to respond effectively to infections and vaccinations [3,4].

In the United States, ethnic minority populations experience disproportionately higher rates of infectious diseases, often due to barriers in accessing healthcare, testing and treatment [5]. Furthermore, variations in immune responses among these groups warrant further investigation, as they may significantly contribute to health disparities [6]. Minority populations may also experience accelerated immunosenescence and increased low-grade systemic inflammation, known as inflammaging [7], leading to an earlier onset of age-related diseases. Emerging evidence indicates racial and ethnic disparities in vaccine responses, with notable differences in antibody production and T cell activation following vaccination [8,9]. Historically, racial and ethnic minorities have been underrepresented in clinical trials, limiting our understanding of immune responses in diverse populations [10]. Therefore, it is imperative to employ ethical and community-focused immunology research to address these disparities in immune function.

In 2020, approximately 6 million Afro-Latino adults resided in the United States, constituting about 2% of the adult population and 12% of the adult Latino population [11]. Afro-Latinos or Black Hispanics are often categorized alongside non-Hispanic Black populations in research studies, while White Hispanics are classified with non-Hispanic Whites [12]. This grouping may obscure significant differences between these demographic groups. Recent studies indicate that the immune systems of Black Hispanics and White Hispanics may exhibit significant variations due to factors such as genetic ancestry, environmental exposures, social determinants of health, and access to healthcare [13,14]. Black Hispanics generally possess higher levels of African ancestry, which are associated with genetic variations that affect cytokine production and inflammatory responses. Additionally, they face an increased risk of conditions such as obesity, type 2 diabetes mellitus (T2DM), hypertension, and cardiovascular disease, all linked to chronic low-grade inflammation and compromised immune function [15]. During the COVID-19 pandemic, both Black and White Hispanics were disproportionately affected compared to non-Hispanic Whites; however, Black Hispanics experienced higher rates of hospitalization and mortality [16].

Our research has previously identified and characterized critical defects in the antibody response to the influenza vaccine among healthy individuals of different ages [17,18], as well as in those with inflammatory conditions and diseases [19]. We have shown that intrinsic defects in immune cell function lead to decreased specific responses to the vaccine. Additionally, we have demonstrated that inflammaging is marked by increased frequencies of pro-inflammatory cell subsets, which are characterized by higher levels of transcripts for pro-inflammatory and senescence-associated markers, and they are hyper-metabolic. This hyper-metabolic status is essential for supporting intrinsic inflammation and immune activation (IA) [20].

This study aims to determine whether influenza vaccine-induced antibody responses differ between White and Black Hispanic individuals in South Florida. We further investigated whether intrinsic inflammatory and metabolic phenotypes of blood-derived B cells contribute to these racial disparities in vaccine responses. Finally, we tested whether in vitro inhibition of metabolic pathways could attenuate the inflammatory phenotype of B cells from Black Hispanic individuals, thereby providing mechanistic insight into immune dysfunction and identifying potential avenues for intervention.

## 2. Materials and Methods

### 2.1. Study Participants

This study used deidentified samples from a prior influenza vaccination study conducted at the University of Miami Miller School of Medicine (UM-MSOM). Study recruitment took place before the recent COVID-19 pandemic, and the study protocol was approved by the UM-MSOM Institutional Review Board (IRB, protocols #20070481 and #20160542). Participants were recruited after providing signed informed consent. Individuals with metabolic disorders, autoimmune diseases, T2DM, cardiovascular diseases, chronic infectious diseases (HIV, malaria), and active malignancies were excluded from the study. Additionally, participants with substance abuse or alcohol abuse issues, those undergoing hormonal replacement therapy, and individuals on immunosuppressant medications or steroids were also excluded. This study included deidentified samples from Hispanic participants who self-identified as White (*n* = 20, age range 42–55 years) or Black (*n* = 12, age range 44–53 years). Within each group, participants were evenly split between females and males.

### 2.2. Influenza Vaccination and Sample Collection

All individuals received the seasonal Trivalent Inactivated Influenza Vaccine, which included the strains A/California/7/2009 (H1N1), A/Victoria/361/2011, and B/Massachusetts/2/2012. They had been vaccinated annually since the 2009–2010 influenza season. Blood samples were collected before vaccination and four weeks (day 28) after vaccination. At enrollment, all participants were free of influenza and respiratory tract infection symptoms, and they did not experience influenza-like symptoms during a six-month follow-up. Blood was collected in Vacutainer CPT tubes, and serum was extracted and cryopreserved at −80 °C. Peripheral blood mononuclear cells (PBMC) were isolated through gradient centrifugation, cryopreserved, and stored at −146 °C. All the cellular immunology assays were performed using PBMC collected before vaccination. On the day of the experiment, PBMC were thawed, and cell viability was assessed using trypan blue counting. PBMC with less than 75% viability were discarded.

### 2.3. Hemagglutination Inhibition (HAI) Assay

The serum response to the influenza vaccine was assessed using the Hemagglutination Inhibition (HAI) assay, a key indicator of vaccine effectiveness [21,22]. This assay measures the ability of specific viruses or their components to cause hemagglutination in red blood cells. Antibodies in serum samples specific to influenza antigens can inhibit this agglutination. To evaluate antibody production in response to the vaccine, paired pre- and day 28 post-vaccination serum samples from the same individual were tested. Serum-inhibiting titers of 1:40 or higher indicate seroprotection against infection. A four-fold increase in titer after vaccination signifies a positive response to the vaccine [21].

### 2.4. B Cell Isolation and Stimulation

Total B cells were isolated from PBMCs using CD19 Microbeads (Miltenyi 130-050-301, Auburn, CA, USA), achieving over 95% purity. The B cells were resuspended in complete medium at a concentration of 10^6^ cells/mL, consisting of c-RPMI (RPMI 1640, Gibco ThermoFisher 11875-093, Waltham, MA, USA) supplemented with 10% FBS (Gibco 10437-028), 100 U/mL Penicillin–Streptomycin (Gibco 15140-122), and 2 mM L-glutamine (Gibco 25030-081). B cells were either left unstimulated or were stimulated for 2 days with 1 µg of CpG (ODN 2006, InVivogen, San Diego, CA, USA) per 10^6^ cells to evaluate lactate secretion in the culture supernatants. In some experiments, cells were stimulated overnight with CpG (1 µg/10^6^ cells) with or without an anti-SLC5A12 antibody (ThermoFisher PA5-110389, diluted 1:500), which blocks lactate.

### 2.5. Lactate Dehydrogenase and Lactate Measurements

Serum Lactate Dehydrogenase (LDH) levels were measured using the Abcam kit ab102526. After 2 days of CpG stimulation, culture supernatants were collected to measure lactate secretion using the L-lactate assay kit (Cayman Chemicals 700510, Ann Arbor, MI, USA).

### 2.6. Flow Cytometry for B Cell Phenotyping and Cell Sorting

Thawed PBMCs (2 × 10^6^/mL) were stained for 20 min at room temperature using the LIVE/DEAD™ Fixable Aqua Dead Cell Stain Kit (ThermoFisher L34966, Waltham, MA, USA) and the following antibodies: anti-CD45 (BioLegend 368540, San Diego, CA, USA), anti-CD19 (BD 555415, San Jose, CA, USA), anti-CD27 (BD 555441), and anti-IgD (BD 555778). This staining allowed for the identification of naive (IgD+CD27−), IgM memory (IgD+CD27+), switched memory (IgD−CD27+), and double negative (DN) memory (IgD−CD27−) B cells [23]. Up to 10^5^ events in the Live/CD45+ cell gate were acquired on an LSR-Fortessa (BD) and analyzed using FlowJo 10.10.0 software. Each experiment included single-color controls for fluorescent compensation and isotype antibodies to establish the gates.

DN memory B cells were sorted using a Sony SH800 cell sorter, employing the LIVE/DEAD™ Fixable Aqua Dead Cell Stain Kit along with fluorochrome-conjugated antibodies: anti-CD45, anti-CD19, anti-CD27, and anti-IgD. Total RNA was extracted from the sorted cell subsets using TRIzol (Thermo Fisher Scientific, Waltham, MA, USA) [23].

### 2.7. Reverse Transcriptase (RT) and Quantitative (q)PCR

Reverse Transcriptase (RT) reactions were conducted using a Mastercycler Eppendorf Thermocycler to synthesize cDNA, following established protocols [24]. In these reactions, 100 ng of total RNA (extracted using Trizol, Ambion) served as the template. For miRNA quantification, RT reactions included specific primers provided with the qPCR primers.

For qPCR, 5 µL of cDNA were utilized. The reactions were carried out in MicroAmp 96-well plates and analyzed on an ABI 7500 machine, (Thermo Fisher Scientific, Waltham, MA, USA) with calculations performed using ABI software V2.3. We determined the cycle threshold (Ct) at which the transcripts of target genes and controls (GAPDH or U6) reached a significant level. The difference in Ct values between the controls and the target gene was calculated as ΔCt. The relative quantity of the target gene was expressed as 2^−ΔCt^ and reported as qPCR values.

All reagents were sourced from ThermoFisher Scientific, Waltham, MA, USA. The Taqman primers used were GAPDH (Hs99999905_m1), Glut1/SLC2A1 (Hs00892681), Hexokinase 1 (HK1, Hs00175976_m1), Hexokinase 2 (HK2, Hs006086_m1), Pyruvate kinase M1/2 (PKM, Hs00761782_s1), LDHA (Hs01378790_g1), LDHB (Hs00929956_m1), PDHX (Hs00185790_m1), SLC5A12 (Hs01054645), TNF (Hs01113624_g1), IL-6 (Hs00985639_m1), IL-8 (Hs00174103_m1), p16^INK4^ (CDKN2A, Hs00923894_m1), p21^CIP1/WAF1^ (Hs00355782_m1), U6 (001973), miR-155 (002623), and miR-16 (000391).

### 2.8. Seahorse Glycolytic Test

B cells were incubated in Seahorse XF DMEM Medium without glucose and then seeded into an extracellular flux analyzer at a concentration of 2 × 10^5^ cells per well for a glycolytic test [23]. The cells were treated with glucose (10 mM), followed by oligomycin (1 μM) and 2-deoxy-glucose (2-DG, 20 mM). Oligomycin inhibits mitochondrial ATP production, shifting energy production to glycolysis, which increases the ECAR and maximum glycolytic capacity. 2-DG, a glucose analog, inhibits glycolysis by binding to hexokinases, leading to decreased ECAR. This confirms that the observed acidification in the test is due to glycolysis.

### 2.9. Statistical Analyses

An unpaired Student’s *t*-test (2-tailed) was conducted to examine group differences. The area under the curve (AUC) was used to compare glycolytic profiles from Seahorse analyses. All graphs were created using GraphPad Prism version 10.1.0.

### 2.10. Supplementary Material

Raw data underlying all figures (including Supplementary Figures S1–S5) can be found in the Excel spreadsheet containing all numerical data in separate sheets.

## 3. Results

### 3.1. Reduced Influenza Vaccine Response in Black Versus White Hispanic Individuals

**Figure 1:**
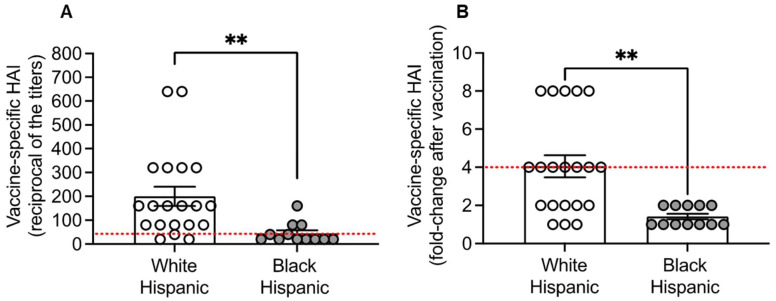
Lower antibody response to flu vaccination in Black Hispanics: Vaccine-specific antibodies were evaluated by HAI. (**A**) Reciprocal of the titers 4 weeks after vaccination. The red dotted line indicates a protective titer of 1:40. (**B**) Fold-change in the reciprocal of the titers after vaccination. The red dotted line indicates seroconversion (4-fold change in titers after vaccination). Mean comparisons between groups were performed by unpaired Student’s *t*-test. ** *p* < 0.01.

Serum samples from White and Black Hispanic individuals were evaluated for seasonal influenza vaccine-specific protective antibodies, measured by the HAI assay, the best correlate of vaccine protection. Results in Figure 1 show a significant decrease in the antibody response to the vaccine in Black Hispanics compared to White Hispanics. We found that both antibody titers (Figure 1A) and fold-changes after vaccination (Figure 1B) were decreased. Conversely, we found no differences in the response to the influenza vaccine when comparing White and Black Non-Hispanic individuals (Figure S1). Consistent with our findings, a previously published study has showed even higher responses to the influenza vaccine in Black Hispanics compared to White Non-Hispanics in both antibody titers and neutralizing antibodies to H1N1 and H3N2 viral strains [25]. This race-related difference was observed only in adults, not in elderly individuals.

### 3.2. Increased Serum Levels of Lactate Dehydrogenase (LDH) in Black Hispanics Compared to White Hispanics

Lactate Dehydrogenase (LDH) is the terminal enzyme in the anaerobic glycolysis pathway, responsible for converting pyruvate into lactate. Elevated serum levels of LDH serve as a biomarker for inflammatory conditions and diseases, as well as indicators of mortality and poor health outcomes in patients with serious inflammatory and immunodeficiency diseases [26]. In this study, we focus on LDH due to its critical role in regulating the intrinsic pathways of cellular metabolism in immune cells, as shown below.

**Figure 2:**
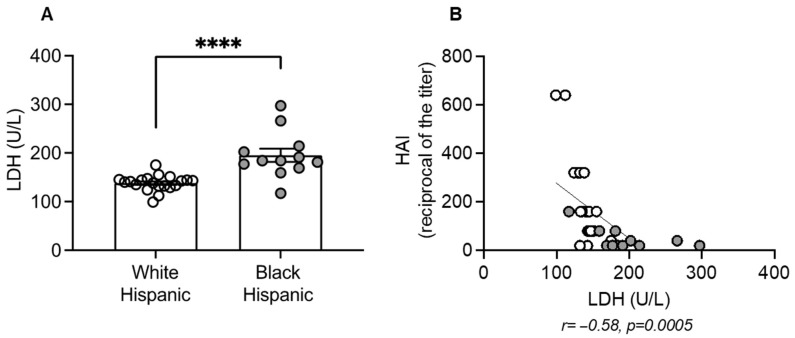
Serum levels of LDH inversely correlated with Flu vaccine-induced antibody response: (**A**) serum samples from the same individuals in Figure 1 were tested in ELISA. Mean comparisons between groups were performed by unpaired Student’s *t*-test. **** *p* < 0.0001. (**B**) Pearson’s r and *p* value are shown below the figure. White symbols, White Hispanics. Gray symbols, Black Hispanics.

We measured serum levels of LDH in our cohort of White and Black Hispanic individuals and found a significant increase in LDH levels in Black Hispanics compared to White Hispanics, as shown in Figure 2A. Additional serum metabolic characterization of the recruited participants is shown in Figure S2. Moreover, serum LDH levels were negatively associated with the serum antibody response to the influenza vaccine, as measured by HAI (Figure 2B).

### 3.3. Increased Secretion of the Metabolite Lactate in B Cells from Black Hispanics

**Figure 3:**
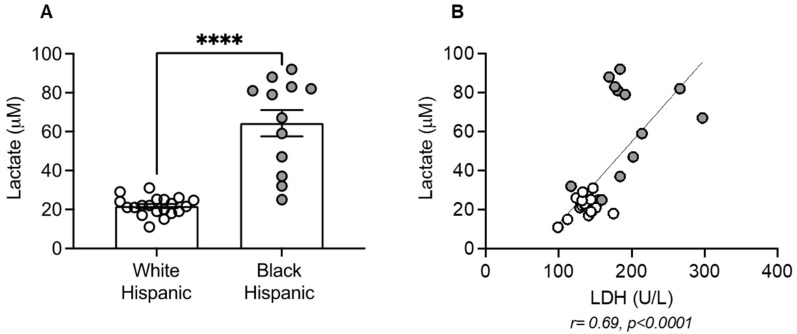
B cells from black Hispanics showed increased lactate secretion: (**A**) B cells isolated from the peripheral blood using magnetic beads were stimulated with CpG (1 µg/10^6^ cells) for 2 days, then culture supernatants were collected and lactate secretion measured by a colorimetric assay. Results show μM of lactate (mean ± SE). Mean comparisons between groups were performed by unpaired Student’s *t*-test. **** *p* < 0.0001. (**B**) Correlation of lactate in culture supernatants and serum LDH. Pearson’s r and *p* value are shown below the figure. White symbols, White Hispanics. Gray symbols, Black Hispanics.

Because of the higher levels of serum LDH in Black Hispanics compared to White Hispanics, we measured the amount of lactate secreted by B cells isolated from these individuals. We first evaluated the kinetics of lactate secretion in unstimulated versus CpG-stimulated B cells from White Hispanic individuals, shown in Figure S3. We found that peak secretion occurs after 48 h of CpG stimulation. Results in Figure 3 demonstrate that B cells from Black Hispanics secrete significantly more lactate after 48 h in culture supernatants compared to those from White Hispanics (Figure 3A). Additionally, levels of lactate in culture supernatants were positively associated with serum levels of LDH (Figure 3B).

### 3.4. Frequencies of DN B Cells, the Most Pro-Inflammatory B Cell Subset, Are Increased in the Blood of Black Hispanics and Are Positively Associated with the Serum Levels of LDH and B Cell-Derived Lactate and Are Negatively Associated with the Antibody Response to the Influenza Vaccine

**Figure 4:**
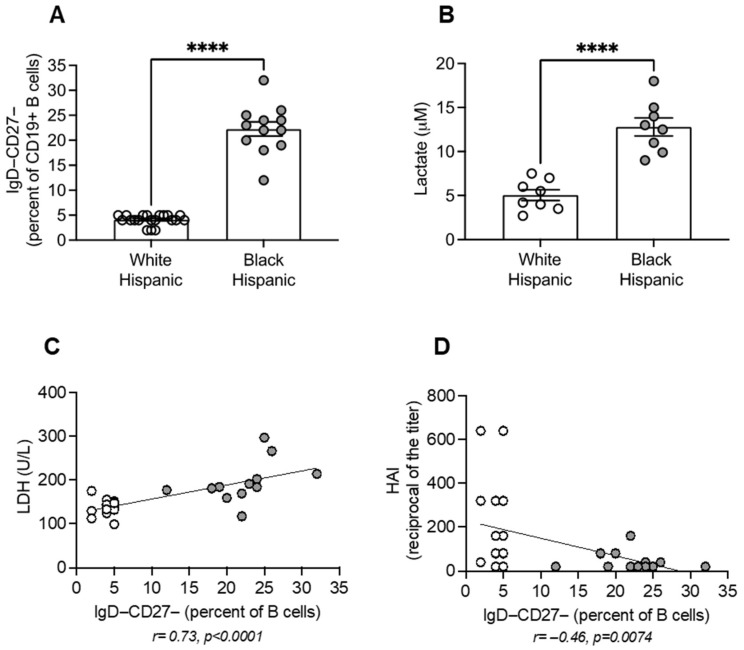
Increased DN B Cells and B Cell-Derived Lactate Associate with Reduced Influenza Vaccine Responses in Black Hispanics: (**A**) PBMC (2 × 10^6^/mL) were stained for 20 min at room temperature with a LIVE/DEAD™ Fixable Aqua Dead Cell Stain Kit and with the following antibodies: anti-CD45, anti-CD19, anti-CD27 and anti-IgD. DN B cells are IgD−CD27−. Results show DN B cell frequencies (mean ± SE). (**B**) Lactate secretion was measured in sorted DN B cells after 2-day CpG stimulation (1 µg/10^6^ cells). Results show μM of lactate (mean ± SE). (**C**) Correlation of DN B cell frequencies and serum LDH. (**D**) Correlation of DN B cell frequencies and influenza vaccine response. Mean comparisons between groups were performed by unpaired Student’s *t*-test. **** *p* < 0.0001. Pearson’s correlation coefficients and *p* values are shown at the bottom of each figure. White symbols, White Hispanics. Gray symbols, Black Hispanics.

Lactate secretion primarily depends on DN B cells, as we have recently demonstrated [23]. We first measured the frequencies of DN B cells in the peripheral blood of White and Black Hispanics. We then sorted DN B cells (from *n* = 8 individuals/group) and measured lactate secretion after 2-day CpG stimulation. The results in Figure 4 show higher frequencies of DN B cells (Figure 4A) that secrete higher amounts of lactate (Figure 4B) in Black Hispanic compared to White Hispanic individuals. As expected, DN B cell frequencies are positively associated with serum LDH (Figure 4C) and negatively associated with the influenza vaccine response measured by HAI (Figure 4D). The composition of the circulating B cell pool in White and Black Hispanic individuals is shown in Figure S4, while the contribution of the naive B cell subset to lactate secretion is in Figure S5. Lactate secretion by IgM memory and switched memory B cells was not evaluated due to limited amounts of sorted cells.

### 3.5. Increased Expression of Metabolic and Inflammatory Markers in B Cells from Black Hispanics

**Figure 5:**
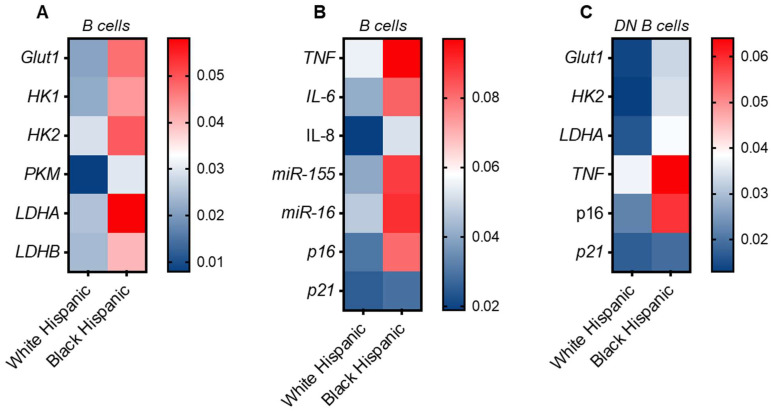
Increased Metabolic–Inflammatory Activity in B Cells from Black Hispanics: B cells, isolated from the peripheral blood using magnetic beads, were left unstimulated, then resuspended in TRIzol, and the RNA was extracted. The expression of metabolic markers involved in oxidative phosphorylation and in anaerobic glycolysis (**A**) or of SASP markers (**B**) was evaluated by qPCR. Results show qPCR values, measures of RNA expression of target genes, relative to the housekeeping genes GAPDH or U6 (for miRs quantification), calculated as 2^−ΔCts^. (**C**) DN B cells were sorted as indicated above, were left unstimulated, then resuspended in TRIzol, and after the RNA was extracted, the expression of metabolic and inflammatory markers was evaluated by qPCR.

We have demonstrated that lactate induces hyper-glycolytic B cells, which are pro-inflammatory [23]. Anaerobic glycolysis and lactate secretion are crucial for the release of markers associated with the senescence-associated secretory phenotype (SASP) [27]. These processes, along with cell and endoplasmic reticulum enlargement, occur with NF-kB signaling and activation, leading to the secretion of pro-inflammatory mediators and pathogenic antibodies. We analyzed the RNA expression of metabolic and inflammatory markers in B cells isolated from the peripheral blood of Black and White Hispanic individuals using qPCR. We measured the expression of Glut1, the primary glucose transporter in human circulating B cells; LDHA, which converts pyruvate into lactate; and PDHX, a component of the pyruvate dehydrogenase (PDH) complex that converts pyruvate into Acetyl CoA, linking glycolysis, the Krebs cycle, and the production of reactive oxygen species. Results in Figure 5 show a higher expression of metabolic transcripts in B cells from Black Hispanics compared to those from White Hispanics, particularly LDHA, consistent with the higher lactate secretion observed (Figure 5A). Due to this increase in LDHA, we evaluated the expression of other markers associated with glucose metabolism and anaerobic glycolysis, such as HK1 and HK2, Hexokinase 1 and 2, that phosphorylate glucose, the initial step in glucose metabolism; Pyruvate kinase M1/2, PKM, that converts phosphoenolpyruvate into pyruvate, the final step in glycolysis; and LDHB, lactate dehydrogenase B, another isoform of lactate dehydrogenase expressed at lower levels in immune cells compared to LDHA. All transcripts for these markers were higher in B cells from Black Hispanics compared to White Hispanics.

We also measured markers of the SASP [pro-inflammatory cytokines (TNF, IL-6, IL-8), pro-inflammatory microRNAs (miRs) (miR-155, miR-16), and cell cycle inhibitors (p16^INK4a^, p21^CIP1/WAF1^)], which were also found expressed at higher levels in B cells from Black Hispanics compared to White Hispanics (Figure 5B). We previously demonstrated that the expression level of metabolic and inflammatory transcripts in B cells negatively correlates with their capacity to respond to exogenous antigens, infections and vaccines, both in vivo or in vitro [18].

We have previously demonstrated that the higher metabolic and inflammatory phenotype of the B cell pool in Black Hispanic individuals primarily depends on DN B cells. We sorted DN B cells from *n* = 8 individuals per group and evaluated the expression of various metabolic and inflammatory transcripts. Results in (Figure 5C) clearly show that the DN B cell subset is predominantly responsible for the expression of these transcripts in the circulating B cell pool.

### 3.6. Increased Glycolytic Activity of B Cells from Black Versus White Hispanic Individuals

**Figure 6:**
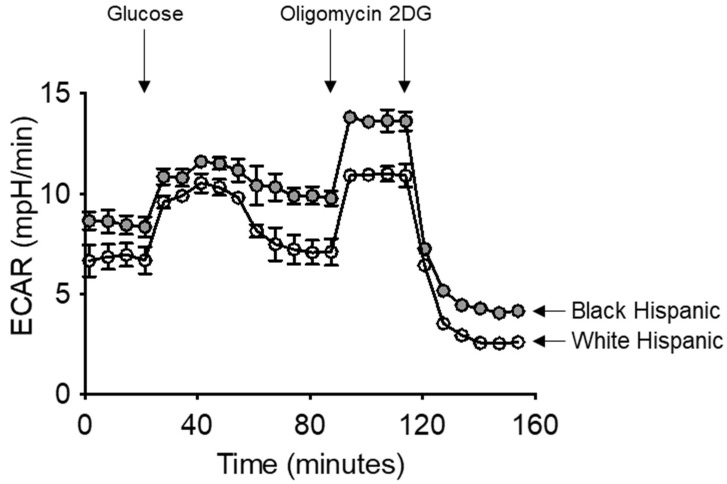
Increased glycolytic activity of B cells from Black Hispanics: B cells, isolated from the peripheral blood using magnetic beads, were left unstimulated and loaded on a Seahorse glycolytic test. Cells were seeded into the wells of an extracellular flux analyzer at the concentration of 2 × 10^5^/well. Calculations of AUC showed significant differences between glycolytic profiles of B cells from Black versus White Hispanic individuals (1442 ± 10 versus 1151 ± 11, respectively, *p* < 0.05). Results are representative of three independent experiments.

The higher glycolytic phenotype of B cells from Black Hispanics was confirmed as follows. B cells from both groups of Hispanic individuals (*n* = 3/group) were loaded on a glycolytic test in Seahorse with sequential addition of glucose, oligomycin and 2-DG. Results in Figure 6 show a hyper-glycolytic phenotype in B cells from Black Hispanic compared to White Hispanic individuals, confirming the previous findings of higher expression of transcripts for Glut1 and enzymes involved in anaerobic glycolysis pathways, and to a lesser extent, PDHX.

### 3.7. Increased Expression of the Lactate Transporter SLC5A12 in B Cells from White and Black Hispanic Individuals

**Figure 7:**
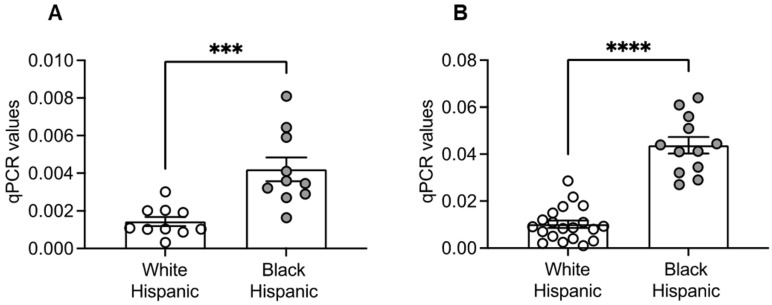
Elevated SLC5A12 Expression in B Cells from Black Hispanics: B cells, isolated from the peripheral blood using magnetic beads, were left unstimulated or they were stimulated overnight with CpG, then resuspended in TRIzol, and the RNA was extracted. The expression of SLC5A12 in unstimulated (**A**) or CpG-stimulated B cells (**B**) was evaluated by qPCR. Results show qPCR values of SLC5A12 relative to the housekeeping gene GAPDH, calculated as 2^−ΔCts^. Mean comparisons between groups were performed by unpaired Student’s *t*-test. *** *p* < 0.001, **** *p* < 0.0001.

This glycolytic phenotype of B cells from Black Hispanic individuals is associated with higher expression of transcripts of the lactate transporter SLC5A12, a low-affinity electroneutral sodium-dependent transporter that catalyzes the transport of many monocarboxylates, such as lactate, across the cell membrane. We measured the expression of SLC5A12 by qPCR in B cells from White and Black Hispanic individuals. Results in Figure 7 show higher expression levels in both unstimulated (Figure 7A) and CpG-stimulated (Figure 7B) B cells from Black compared to White Hispanics, consistent with the earlier findings of increased lactate secretion in B cells from Black versus White Hispanics.

### 3.8. Inhibition of the Lactate Transporter Significantly Decreases the Expression of RNA Transcripts for Metabolic and Inflammatory Markers in B Cells from Black Hispanics

**Figure 8:**
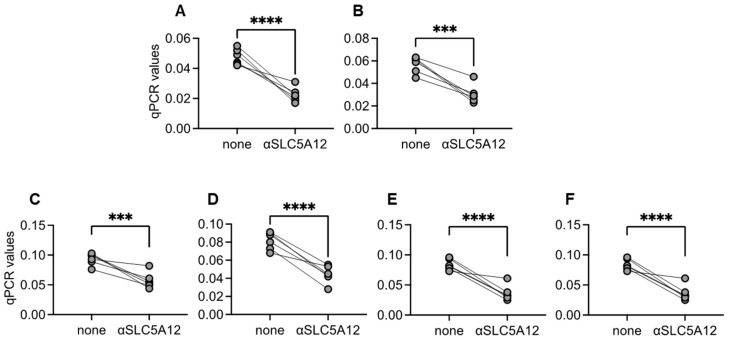
Blocking Lactate Transport Attenuates Metabolic and Inflammatory Gene Expression in B Cells from Black Hispanics: B cells, isolated from the peripheral blood of Black Hispanic individuals using magnetic beads, were stimulated overnight with CpG (1 µg/10^6^ cells), in the absence or presence of anti-SLC5A12 antibody (1:500 diluted). Then, cells were resuspended in TRIzol, the RNA was extracted, and the expression of Glut1 (**A**), LDHA (**B**), TNF (**C**), IL6 (**D**), p16^INK4a^ (**E**) and p21^CIP1/WAF1^ (**F**) were evaluated by qPCR. Mean comparisons between groups were performed by paired Student’s *t*-test. *** *p* < 0.001, **** *p* < 0.0001.

We next evaluated the effect of an anti-SLC5A12 neutralizing antibody, which blocks the lactate transporter SLC5A12 and anaerobic glycolysis/ECAR, on the expression of RNA transcripts for metabolic and pro-inflammatory markers in B cells from Black Hispanics. Results in Figure 8 show that in vitro treatment with the anti-SLC5A12 antibody significantly decreases RNA levels of Glut1, LDHA, TNF, IL-6, p16^INK4a^, and p21^CIP1/WAF1^) in B cells from Black Hispanic individuals.

## 4. Discussion

In this study, we identified novel mechanisms by which metabolic dysfunction amplifies immune cell defects and contributes to reduced production of protective antibodies following influenza vaccination in Black compared to White Hispanic individuals living in South Florida. To our knowledge, these findings are the first to demonstrate racially distinct humoral responses within a Hispanic population, with Black Hispanics exhibiting significantly lower post-vaccination antibody levels. This diminished antibody response was linked to an intrinsic pro-inflammatory phenotype in blood-derived B cells, supported by altered metabolic activity. These results underscore the importance of understanding immune differences between Black and White Hispanics, as such insights are critical for developing targeted strategies to improve vaccine responsiveness and overall health outcomes in this diverse and understudied group.

Our previously published findings have identified critical defects in the immunologic cascade related to specific antibody responses to the influenza vaccine in healthy individuals across different ages [28], as well as in those affected by obesity [29], T2DM [19] and chronic viral infections such as HIV [20]. All these conditions are characterized by inflammaging and persistent IA, which contribute to dysfunctional immune cells and reduced immunity to infections and vaccines, as reviewed in [18]. Inflammaging is also associated with metabolic changes, including mitochondrial dysfunction [30,31], decreased insulin sensitivity [32,33], increased nutrient uptake [34] and elevated blood levels of metabolites such as lactate which in turn support the secretion of inflammatory products and exacerbate inflammaging [35].

Although seasonal influenza vaccination effectively reduces the burden of illness, transmission, hospitalizations, and death, vaccination rates have historically been lower among Black individuals compared to White individuals, despite a higher burden of illness in Black communities [36,37]. Moreover, both immunogenicity and efficacy (production and duration) of vaccine-specific protective immunity appear to be diminished in minority populations facing health disparities, increasing their vulnerability to infection and its complications [38,39]. Different racial groups may also exhibit varying prevalence rates of metabolic disorders such as obesity, diabetes, and chronic inflammation, as well as disparities in nutritional status and microbiome diversity, all of which can impact immune responses to vaccines. Studies indicate that populations with African ancestry, including Black Hispanics, often show lower antibody titers after vaccination compared to those of European ancestry, such as White Hispanics [25,40]. However, the underlying immune mechanisms remain unclear. Many Black individuals have lower vaccination rates compared to White individuals, a disparity influenced by factors such as healthcare access, insurance coverage, lack of accurate information about vaccine efficacy, and socio-economic status [41]. However, the immune response to the influenza vaccine in racially diverse Hispanics remains largely unknown.

Our study aims to identify metabolic pathways involved in regulating humoral immunity in racially diverse Hispanics, with the goal of developing innovative therapeutic interventions to enhance the response to the influenza vaccine in this vulnerable population. The role of the individual’s metabolic status, as well as the metabolic pathways/markers of B cells and their implications on antibody responses to influenza vaccination in racially diverse Hispanics is unknown. Our results herein show that in Black Hispanics, metabolic inflammation exacerbates B cell defects due to dysregulated metabolic reprogramming, primarily marked by increased anaerobic glycolysis and lactate secretion. B cells with a higher metabolic phenotype, as observed in Black Hispanics, are associated with higher intrinsic inflammation and a decreased ability to generate protective antibody responses. Our results further highlight the role of metabolic abnormalities of immune cells in Black Hispanics compromising their influenza vaccine effectiveness. We believe that the identification of metabolic factors that are correlated with vaccine responses and B cell function in racially diverse Hispanics will help to develop targeted interventions to enhance immune responses in racial health disparities among those who do not respond adequately to the influenza vaccine.

This study has several limitations. Its observational design prevents the establishment of causality between B cell metabolic reprogramming, inflammatory phenotypes, and reduced influenza vaccine responses in Black Hispanics. Although our in vitro experiments indicate that blocking metabolic pathways may decrease B cell inflammation, these results are preliminary and require confirmation through in vivo studies. Additionally, various confounding factors including socioeconomic status, access to healthcare, vaccination history, environmental exposures, nutrition, and unmeasured influences such as microbiome composition, chronic stress, and comorbid conditions may also affect immune responses. Furthermore, the modest sample size, although matched for age and sex, limits the generalizability of our findings to the wider Hispanic population. Future research should involve larger, multi-site cohorts with comprehensive sociodemographic and clinical data to validate these results and clarify the relative impacts of biological and social determinants, ultimately guiding interventions aimed at reducing disparities in vaccine-induced immunity.

## 5. Conclusions

In conclusion, our findings demonstrate a lower effect of influenza vaccination in Black Hispanic individuals, a minority population disproportionately affected by chronic diseases, resulting in a higher burden of influenza infection and increased rates of hospitalization and death. Yearly influenza vaccination is highly recommended for these individuals to boost specific antibody titers. Our results also suggest novel strategies for therapeutic interventions using metabolic modifiers to block pathways of B cell intrinsic inflammation and improve protective humoral immunity not only in Black Hispanics but also in other vulnerable populations with diminished vaccine responses

## Data Availability

The original contributions presented in this study are included in the article/Supplementary Materials. Further inquiries can be directed to the corresponding author(s).

## Supplementary Materials

The following supporting information can be downloaded at: https://www.mdpi.com/article/10.3390/vaccines13090938/s1, Figure S1: Vaccine-specific antibodies were evaluated by HAI; Figure S2: Metabolic characterization of serum samples from White and Black Hispanic individuals; Figure S3: Kinetics of lactate secretion by CpG-stimulated B cells; Figure S4: Gating strategies to identify B cell subsets; Figure S5: Lactate secretion by B cell subsets from White and Black Hispanic individuals.
