# Supplementary material for: Metabolic Regulation of Influenza Vaccine Responses in Racially Diverse Hispanics
Source: Vaccines (Basel). 2025 Sep 2;13(9):938. doi: 10.3390/vaccines13090938 (PMC12474446; doi:10.3390/vaccines13090938)
Supplement: Supplementary file 1 [file vaccines-13-00938-s001.zip › vaccines-3779049-supplementary.pdf]

Supplementary Figures

Fig. S1.

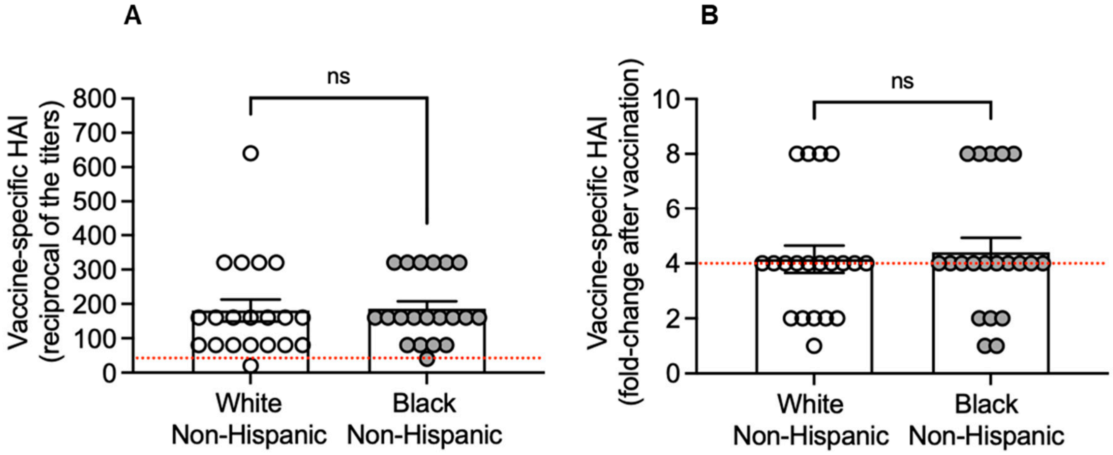

Fig. S1. Vaccine-specific antibodies were evaluated by HAI. (A) Reciprocal of the titers 4 weeks after vaccination. The red dotted line indicates a protective titer of 1:40. (B) Fold-change in the reciprocal of the titers after vaccination. The red dotted line indicates seroconversion (4-fold change in titers after vaccination). Mean comparisons between groups were performed by unpaired Student's t test. ns: not significant.

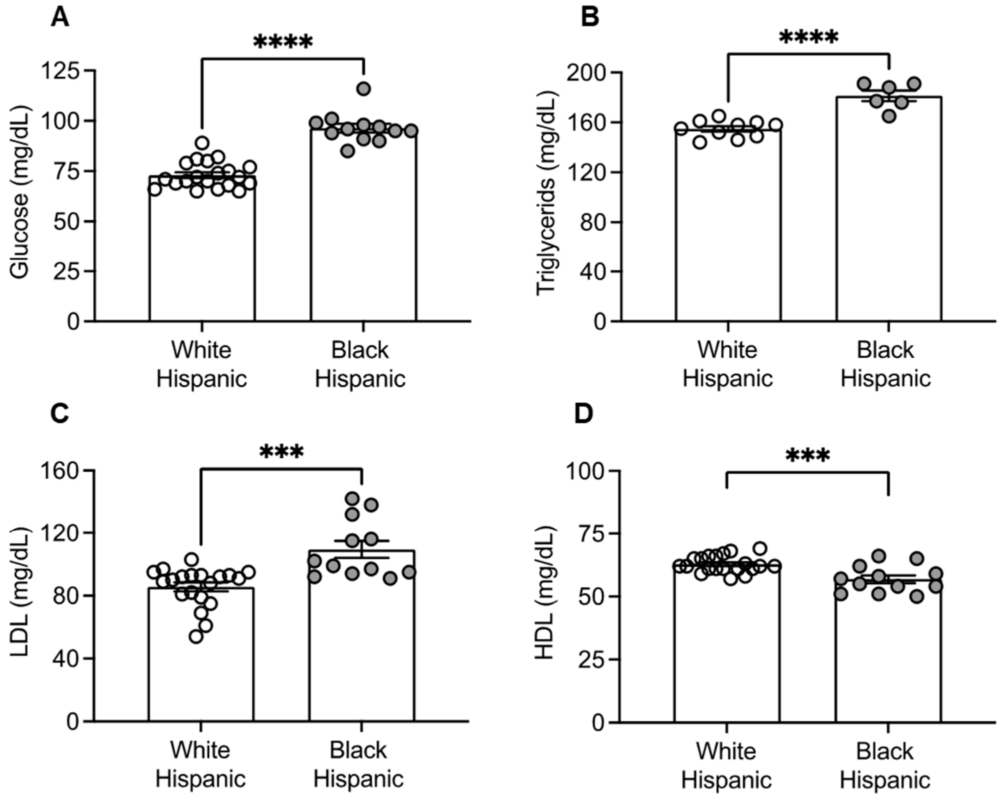

Fig. S2. Metabolic characterization of serum samples from White and Black Hispanic individuals. Mean comparisons between groups were performed by unpaired Student's t test. ***p<0.001, ****p<0.0001.

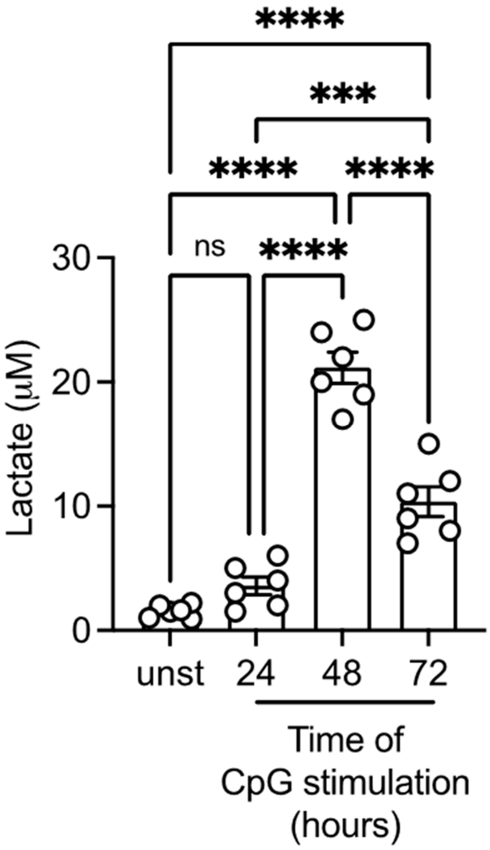

Fig. S3. Kinetics of lactate secretion by CpG-stimulated B cells. B cells from White Hispanic individuals were isolated from the peripheral blood using magnetic beads. Lactate secretion in culture supernatants of unstimulated or CpG-stimulated B cells (24-72 hours) measured by ELISA, means±SE. Mean comparisons among the groups were measured by one-way ANOVA (multiple comparisons). ***p<0.001, ****p<0.0001, ns: not significant.

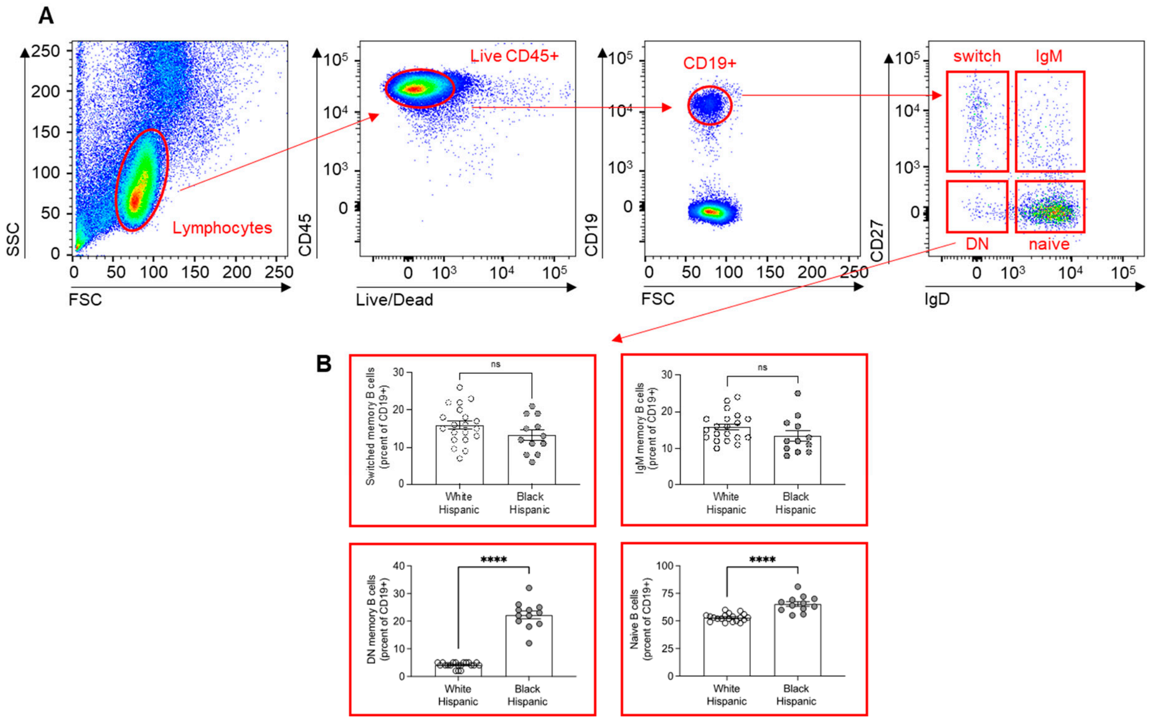

Fig. S4. Gating strategies to identify B cell subsets. (A) Representative dot blots from one White Hispanic individual. PBMC ($2x10^6$/ml) were stained for 20 min at room temperature with Live/Dead kit, and with anti-CD45, anti-CD19, anti-CD27 and anti-IgD fluorochrome-conjugated antibodies to evaluate naive (IgD+CD27-), IgM memory (IgD+CD27+), switched memory (IgD-CD27+), and DN (IgD-CD27-) B cells. (B) Cumulative results from 20 White and 12 Black Hispanic individuals. Mean comparisons between groups were performed by paired Student's t test (two-tailed). ****p<0.0001.

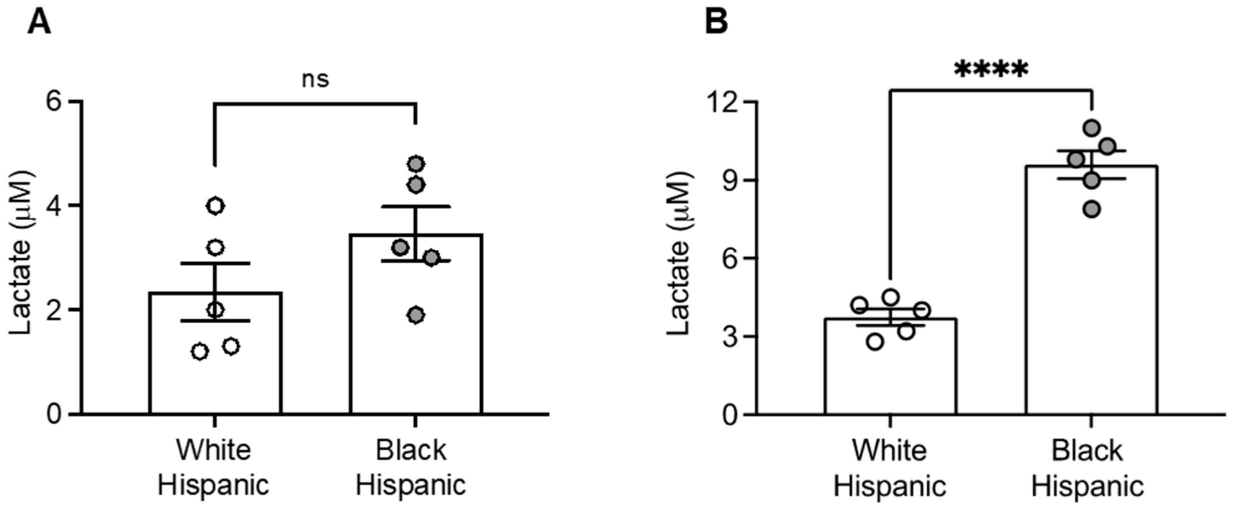

Fig. S5. Lactate secretion by B cell subsets from White and Black Hispanic individuals. Lactate secretion in culture supernatants of stimulated naïve (A) and DN (B) B cells was measured by ELISA. Naïve and DN B cells were stimulated with CpG (1 μg/10^6 cells) in the presence of an AffiniPure F(ab')$_2$ fragment of goat anti-human IgG+IgM (2 μg/10^6 cells). Mean comparisons between groups were performed by unpaired Student's t test. ns=not significant, ****$p<0.0001$.
